# Effect of genotyping error in model-free linkage analysis using microsatellite or single-nucleotide polymorphism marker maps

**DOI:** 10.1186/1471-2156-6-S1-S153

**Published:** 2005-12-30

**Authors:** Cheryl L Thompson, Dan Baechle, Qing Lu, George Mathew, Yeunjoo Song, Sudha K Iyengar, Courtney Gray-McGuire, Katrina AB Goddard

**Affiliations:** 1 Department of Epidemiology and Biostatistics, Case Western Reserve University, Cleveland, OH, USA; 2 Department of Mathematics, Southwest Missouri State University, Springfield, MO, USA

## Abstract

Errors while genotyping are inevitable and can reduce the power to detect linkage. However, does genotyping error have the same impact on linkage results for single-nucleotide polymorphism (SNP) and microsatellite (MS) marker maps? To evaluate this question we detected genotyping errors that are consistent with Mendelian inheritance using large changes in multipoint identity-by-descent sharing in neighboring markers. Only a small fraction of Mendelian consistent errors were detectable (e.g., 18% of MS and 2.4% of SNP genotyping errors). More SNP genotyping errors are Mendelian consistent compared to MS genotyping errors, so genotyping error may have a greater impact on linkage results using SNP marker maps. We also evaluated the effect of genotyping error on the power and type I error rate using simulated nuclear families with missing parents under 0, 0.14, and 2.8% genotyping error rates. In the presence of genotyping error, we found that the power to detect a true linkage signal was greater for SNP (75%) than MS (67%) marker maps, although there were also slightly more false-positive signals using SNP marker maps (5 compared with 3 for MS). Finally, we evaluated the usefulness of accounting for genotyping error in the SNP data using a likelihood-based approach, which restores some of the power that is lost when genotyping error is introduced.

## Background

Genotyping errors occur in large datasets. Errors can arise for many reasons including data entry, technician, or assay errors. As we continue to genotype large numbers of microsatellite (MS) and single-nucleotide polymorphism (SNP) markers, we must consider the effect of these genotyping errors on our ability to detect or find genes. Although several previous studies have shown that genotyping error can reduce the power to detect linkage [1,2], a comparison of the effect of genotyping error on MS marker maps vs. SNP marker maps has not been performed.

Genotyping error can be divided into two types, those that do and do not result in Mendelian inconsistencies. Genotyping errors that result in Mendelian inconsistencies can often be detected using a single marker, such as the segregation of five or more alleles in a nuclear family. Linkage programs that detect and remove Mendelian inconsistent genotyping errors are available (e.g., SIBPAL [3], SIMWALK2 [4], PEDCHECK [5], etc). However, many genotyping errors will be consistent with Mendelian inheritance. Methods to detect genotyping errors that are consistent with Mendelian inheritance include identification of tightly linked double recombinants, which relies on multipoint marker information [6], and distortions of Hardy-Weinberg proportions [7-11]. In addition, likelihood-based approaches are available, which do not aim to detect and remove the genotyping errors, but instead, incorporate the possibility of genotyping error into the computation of the likelihood [4,12-14]. However, these methods typically identify fewer than 50% of the genotyping errors that are consistent with Mendelian inheritance.

The proportion of genotyping errors that are consistent with Mendelian inheritance, and thus less easily detectable using current methods, depends on the type of marker and the family structure being evaluated. In the extreme case, all genotyping errors are Mendelian consistent in situations with biallelic markers and sibship data without parents [12]. Although genotyping error rates may be lower for SNPs than MS markers on a per marker basis, the much larger number of markers that will typically be used for SNP maps means that on an absolute scale there may be more error in SNP maps. What remains unclear is the effect of these errors on the linkage results. In this paper, we use the Genetic Analysis Workshop 14 (GAW14) simulated dataset to evaluate the effect of genotyping error on MS and SNP marker maps. We show that genotyping errors that are consistent with Mendelian inheritance are difficult to detect as double recombinants using multipoint information, and thus are likely to remain in the analysis. This may have a greater impact on SNP marker maps compared to MS marker maps, because SNP genotyping errors are more likely to be consistent with Mendelian inheritance and, as we show, MS errors are easier to detect with this method. In addition, we show that although the simulated SNP marker map resulted in greater power to detect true linkage signals, in the presence of genotyping error there was also an increase in the number of false-positive signals.

## Methods

### Simulation of data

Because we were interested in sib pairs, we used the GAW14 simulated data from the three nuclear family populations (Aipotu, Danacaa, and Karngar). We combined the three populations to increase our power and used all 100 replicates. Parental information was removed to evaluate sib pairs without known parental genotypes. MS marker maps were simulated with 7-cM marker spacing, and SNP marker maps were simulated with 3-cM marker spacing. The genetic model for affected status is described elsewhere. Random genotyping error was simulated at error rates of 0.14% and 2.8%, which were selected to represent typical error rates for SNP and MS datasets, respectively. To simulate genotyping error for the SNP data, genotypes were randomly chosen for replacement at the specified error rate, and one of the alleles was selected randomly and changed to the other allele. To simulate genotyping error for the MS data, genotypes and alleles were randomly selected for replacement as above; however, the allele was replaced by one of the alleles adjacent in size (i.e., either one more or one fewer repeats) to mimic laboratory conditions.

### Detection of Mendelian consistent genotyping error as double recombinants

Mendelian consistent genotyping error is not detectable based on information from a single marker. However, this type of genotyping error may appear as a double recombinant in multipoint analysis [15], which could be detected as a large change in the identity-by-descent (IBD) sharing on both sides of a particular location. In addition, we can identify which individual within the family had the genotyping error by identifying the common individual among all pairs of individuals with large changes in the IBD sharing. We obtained estimates of multipoint IBD sharing among siblings using GENIBD [3]. To detect double recombinants, we examined the difference in the estimates for sharing 0 and 2 alleles IBD between the current marker and the one preceding it (δ_pre_) and the current marker and its successor (δ_post_) for each pair of individuals at each marker. If the absolute values of δ_pre _and δ_post _exceeded the same predetermined cutoff, δ, for two or more sib pairs that included the same individual, the marker was deemed to be the site of a double recombinant for that individual. This means that double recombinants cannot be detected for pedigrees with a single sib pair or at the ends of the chromosome by our definition. The false- positive and false-negative rates were computed separately for the MS and SNP markers based on knowledge of the simulated errors. The Shannon information content (SIC) was computed using MLOD [3] to evaluate the error rates as a function of the SIC.

### Evaluation of power and type I error for MS and SNP marker maps

To evaluate the power and type I error rates, we performed model-free linkage analysis separately for the MS and SNP data using the w4 option in SIBPAL [3]. As implemented in SIBPAL, the Haseman-Elston method regresses a weighted combination of the squared trait difference and squared mean-corrected trait sum on the estimated proportion of alleles shared IBD. The weights are chosen to be proportional to the inverse of the residual variances of the squared differences and sums. To compute the power, we defined a true positive as a signal that exceeded the given threshold, and that had a peak location within 20 cM of the true location per the simulation answers. To evaluate the type I error rate, a false positive was defined as a signal that exceeded the given threshold, that had a peak location more than 40 cM from the true location of a disease gene, and that was more than 20 cM from any other peak location, i.e., there could be more than one false positive on a chromosome.

### Evaluation of likelihood-based approach to account for genotyping error

The loss in power that results from genotyping error can be minimized using likelihood-based approaches to account for the genotyping error in the linkage analysis [4,12]. We incorporated this approach into SIBPAL by modifying the marker penetrances such that the probability that the observed genotype is the correct genotype is 1-ε, and the probability that it is any other genotype is ε/(n-1), where n is the total number of genotypes for that locus. We reanalyzed the SNP simulated data with GENIBD and SIBPAL using the correct error rate that was simulated (i.e., with ε = 0.0014 and 0.028), and evaluated the power and type I error rate as described above.

## Results

### Detection of genotyping error as double recombinants

**Figure 1:**
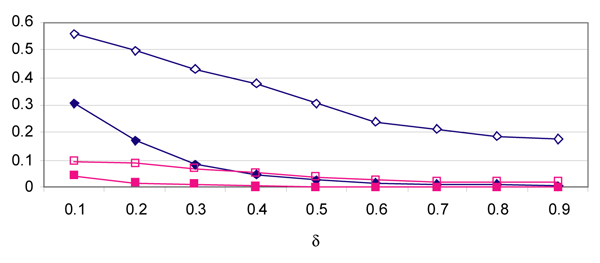
**True positive and false positive rates of the method to detect double recombinants versus δ**. True positive rates are represented by blue diamonds and false positive by pink squares. Rates are plotted versus δ (difference in IBD sharing between adjacent markers) for MS (open symbols) and SNP (closed symbols) marker maps.

For the MS marker map, on average 35.6% of the genotyping errors were detected as Mendelian inconsistencies, and removed from the analysis, whereas none (0%) of the genotyping errors were identified as Mendelian inconsistencies for the SNP marker map. Therefore, under the most rigorous condition (δ = 0.9), 53.1% of the genotyping errors were detected for the MS marker map, whereas only 2.4% of the genotyping errors were detected for the SNP marker map. We then utilized sib-pair IBD sharing estimates to identify Mendelian consistent genotyping errors as double recombinants, detected as large changes in IBD sharing estimates from neighboring markers on both sides of a particular location. Figure 1 shows the true-positive and false-positive rates of this method averaged across all markers and all families, for both the MS and SNP marker maps with a 2.8% simulated error rate. In general, the method performed poorly with high false-positive rates at low values of δ, and low true-positive rates at high values of δ.

**Figure 2:**
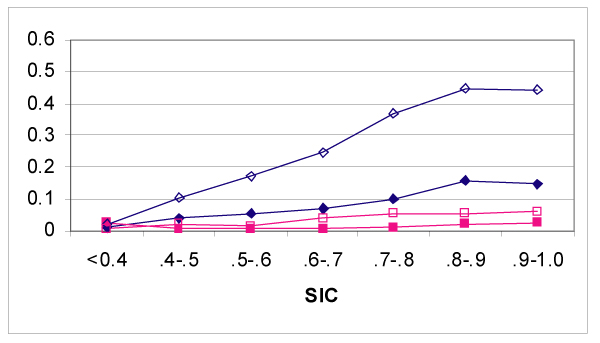
**True and false positive rates by SIC**. True positive rates are represented by blue diamonds and false positive by pink squares. Rates are plotted by Family Specific Shannon Information Content (δ = 0.5) for MS (open symbols) and SNP (closed symbols) marker maps.

The ability to detect Mendelian consistent genotyping errors may depend on factors such as the sibship size and the marker information content. Therefore, we explored the true- and false-positive rates of our method based on sibship size and family specific SIC (Figure 2). As expected, Mendelian consistent genotyping error was more frequently detected under circumstances with more complete information (e.g., larger sibship sizes and higher SIC). However, even with the same SIC, Mendelian consistent genotyping error was more frequently detected by this method of finding double recombinants using the MS marker map (e.g., true-positive rate of 0.45 for SIC between 0.9–1.0) compared to the SNP marker map (e.g., true-positive rate of 0.20 for SIC between 0.9–1.0) with similar values for the false-positive rate.

### Effect of genotyping error on power and type I error

For all levels of genotyping error, the SNP marker map was more powerful than the MS marker map for detecting linkage (Table 1). However, the SNP marker map also had a higher false-positive rate in the presence of genotyping error compared to the MS marker map (Table 2), suggesting that a different threshold should be employed for declaring significant evidence of linkage for the two marker maps. Marker-specific genotyping error rates are expected to be lower for SNP markers compared to MS markers, so a more fair comparison might be to compare the power for the SNP marker map at the 0.14% error rate to the MS marker map at the 2.8% error rate. However, even under this circumstance the SNP marker map has slightly higher power to detect significant evidence of linkage (*p*-value < 2.2 × 10^-5^) compared to the MS marker map (75% vs. 67%), while the false-positive rates are more similar (5 for SNP and 3 for MS).

Genotyping error decreases the power to detect linkage for both types of marker maps. As shown in Figure 2, the type I error rate dramatically increased as the genotyping error rate increased for the SNP marker map, while remaining fairly constant for the MS marker map. This implies that use of a two-stage approach (i.e., genome scan using MS markers and follow-up using SNPs) would be a better overall strategy in terms of reducing cost and effort for fine-mapping.

### Results from the likelihood-based approach to account for genotyping error

After using the likelihood approach to account for genotyping error, we found that for the SNP marker map, there was very little improvement in either the true- or false-positive rate at the 0.14% error level. At the higher 2.8% error rate, while the true-positive rate remained fairly constant, there was a significant improvement in the false-positive rate. However, this error rate is probably much higher than is found in more recent genotyping.

Mendelian consistent genotyping errors were more easily detected with our method for the MS marker map than the SNP marker map, even at the same marker information content. This suggests that when the error is corrected or accounted for using the likelihood-based approach, a greater amount of power will be restored for the MS marker map than the SNP marker map.

## Conclusion

The effect of genotyping error on linkage analysis for SNP vs. MS marker maps is similar if you compare the power for the SNP marker map at the 0.14% error rate to the MS marker map at the 2.8% error rate. The SNP marker map has slightly higher power to detect significant evidence of linkage compared to the MS marker map and similar false-positive rates.

To make a fair comparison between the MS and SNP performances in our method to detect double recombinants, we need to compare the true-positive rate at similar false positive rates. A δ = 0.9 for the MS gives approximately the same false-positive rate (1.97%) as a δ = 0.2 for SNPs (1.70%). At these deltas, the true-positive rates are also very similar, 17.5% for MS and 16.9% for SNPs. From this, we can conclude that our method for detecting double recombinants performs very comparably for MS and SNP markers, but to achieve those error rates, different cutoffs for delta should be used depending on the type of marker.

On the whole, our method to detect double recombinants resulted in more false positives than true positives. We conclude that it is not practical to detect genotyping errors as double recombinants through large changes in IBD sharing. Most of the situations that were identified through this method as a genotyping error were actually false positives. These results are consistent with the results of the method implemented in SIMWALK2 [4,6]. Badzioch [6] reports a maximum error detection rate of 50% and notes that as many as 70% of the errors detected were false positives.

Another issue with this method of identifying double recombinants is that if a single recombinant occurs in both siblings, and the recombinant occurs on one side of a given marker in the first sibling and on the other side of that marker in the second sibling, the two siblings could in fact share an additional allele IBD for only that single marker. This method would have no way of distinguishing between a double recombinant and two single recombinants occurring in almost the exact same chromosomal region in two individual siblings and may account for some of the false positives that have been encountered. However, as the field moves toward using SNP marker maps, the marker density will increase and the chance of this happening will decrease. It would be interesting to look at the effect of the density of a map given the same type of markers on the false positive rate of this method.

Genotyping errors have always been a problem in linkage analysis. In this paper we evaluated error levels of 0.14% and 2.8%, which are within the bounds of realistic genotyping error rates [5,16]. Methods to find and correct Mendelian consistent genotyping errors generally rely on detecting double recombinants and assuming them to be genotyping errors. Likelihood-based extensions to linkage analysis to maximize the power in the presence of undetected genotyping error have been made. As genotyping error rates shrink and cost of genotyping decreases, the importance of dealing with genotyping error will fade. However, the current reality for many datasets is that there are higher undetected genotyping error rates. Error rates as low as 1% can significantly affect the power of multipoint linkage analysis [1,2]. Attempting to remove them by looking for double recombinants via sharp changes in IBD sharing is not a reasonable solution.

## Abbreviations

GAW14: Genetic Analysis Workshop 14

IBD: Identity-by-descent

MS: Microsatellite

SIC: Shannon information content

SNP: Single-nucleotide polymorphism

## Authors' contributions

CLT ran some of the analyses, carried out a literature search, and the drafting of this manuscript. DB assisted with the initial design and wrote the program to detect errors. QL simulated the errors and assisted in the writing. GM ran some of the analyses and assisted in the manuscript revision. YS assisted with the analysis. SKI contributed to the initial conception and design and provided critical revisions of the manuscript. CG-M assisted with the initial conception and design, analysis, and statistical interpretation. KABG contributed to the initial conception and design, analysis interpretation and the writing of this article. All authors approved the final version of the manuscript.

## Figures and Tables

**Table 1:** True-positive rate (%)

			Uncorrected				Corrected^a^	
				
	No error		0.14% error		2.8% error		0.14% error	2.8% error
					
Threshold	MS	SNP	MS	SNP	MS	SNP	SNP	SNP
7.4 × 10^-4^	90	94	89	93	89	90	91	90
2.2 × 10^-5^	74	75	71	75	67	68	70	68
1.0 × 10^-6^	50	59	48	60	42	48	53.5	50.5

^a^Likelihood-based approach was used to account for genotyping error in the analysis.

**Table 2:** Number of false-positive signals

			Uncorrected				Corrected^a^	
				
	No error		0.14% error		2.8% error		0.14% error	2.8% error
					
Threshold	MS	SNP	MS	SNP	MS	SNP	SNP	SNP
7.4 × 10^-4^	35	35	34	37	34	52	37	38
2.2 × 10^-5^	3	4	3	5	3	10	6	6
1.0 × 10^-6^	1	1	1	0	0	1	2	2

^a^Likelihood-based approach was used to account for genotyping error in the analysis.
